# Top-down and bottom-up attention for joint pattern classification and reconstruction

**DOI:** 10.1371/journal.pone.0351985

**Published:** 2026-07-01

**Authors:** Ricardo A. Veiga, Bonifacio Silvano Zanutto

**Affiliations:** 1 Facultad de Ingeniería, Universidad de Buenos Aires, Instituto de Ingeniería Biomédica, Buenos Aires, Argentina; 2 Laboratorio de Biología del Comportamiento, Instituto de Biología y Medicina Experimental (IBYME-CONICET), Buenos Aires, Argentina; Islamia University of Bahawalpur: The Islamia University of Bahawalpur Pakistan, PAKISTAN

## Abstract

We introduce a recurrent inference framework for the Classification and Reconstruction of Overlapping Patterns (CROP) in mixtures formed by overlapping two patterns drawn from the same distribution. The framework alternates between bottom-up classification and top-down generative reconstruction within an iterative inference procedure. At each iteration, the method estimates the most likely class present in the mixture, reconstructs the corresponding signal using a conditional generative model, and applies a mask to isolate that component. This classification-guided reconstruction progressively separates the overlapping signals while also producing their class labels. The objective is therefore to iteratively separate and classify the overlapping patterns rather than perform general blind source separation. An important feature of the framework is that the generative model can be trained using only clean samples, without requiring paired mixed–clean training data. The iterative procedure implicitly implements a form of attention in which saliency- and priority-driven estimates guide the masking and reconstruction of individual patterns. Experimental results on mixtures of handwritten digits show that the proposed framework can successfully separate and classify overlapping patterns through this iterative classification–reconstruction process.

## Introduction

In visual processing, the separation and classification of similar overlapping patterns present a significant challenge, with applications across various domains. For instance, in image processing, overlapping signals originating from the same process are common. X-ray imaging is often used to detect suspected shorted wire bonds in microelectronics, where overlapping frequently occurs within the image field of view [1]. Similarly, X-ray images are employed in medical diagnostics to detect overlapping bones, or in security inspections to identify prohibited items, where substantial overlap is prevalent [2]. A related challenge arises in the classification and reconstruction of spatially overlapping phase images, such as those encountered in electron microscopy of cultured cells or thin tissue sections [3]. Despite the difficulties faced by traditional artificial visual processing systems, the human brain can efficiently separate and recognize overlapping patterns.

Attention, a critical cognitive function, plays a central role in daily activities such as reading, listening, navigating the environment, avoiding distractions, selecting objects, and specifically, classifying and filtering overlapping patterns. Researchers in psychology have extensively studied processes like prediction and focusing, which enable individuals to prioritize target variables —such as spatial locations, features, and objects— and attend specifically to those targets [4,5]. Even salient sensory stimuli, such as a bright light or a loud sound, can capture attention [6].

Over the past decades, several models of attention have been developed; however, some lack explicit biophysical mechanisms or detailed representations of neural circuits [7,8]. Although other researchers have pursued more biologically inspired approaches, it remains difficult to distinguish brain signals related to action intention from those linked to perceptual prioritization. Action intentions initiate subsequent actions, while perceptual prioritization enhances specific features of target items through top-down and bottom-up processes, respectively [4,9]. In most studies, these processes are commonly classified as goal-driven attention (intentional actions by the agent) and stimulus-driven attention (responses to salient stimuli). Moreover, incorporating the generic notion of “bias” to explain behavioral selection [10] suggests that task representations establish top-down selection bias, physical salience generates bottom-up bias (often represented as a saliency map), and biases acquired through learning from past experiences further shape attentional selection. Modulatory effects in perceptual and attention-control areas also prioritize relevant stimuli via biased competition, while filtering out irrelevant information [11].

Carpenter and Grossberg [12] introduced top-down attentional and matching mechanisms as key elements in self-stabilizing learning. Since then, numerous architectures have been proposed to model attentional behavior. For example, Al-Tahan et al. [13] interpreted visual reconstruction as a feedback process within the ventral pathway, while Ahn et al. [9] proposed the Object Reconstruction-guided Attention (ORA) model, which uses top-down signals to guide spatial attention.

Despite these advances, efficiently separating and classifying similar overlapping patterns in an interpretable manner remains a long-standing challenge [3,9,14]. Existing approaches typically address only part of the problem or introduce practical limitations. Many models rely on complex architectures or training procedures that are difficult to adapt, while others either perform classification without explicit reconstruction or require extensive tuning and large annotated datasets. In particular, a common assumption is access to paired samples of mixed and clean signals $\left(\tilde{x},x\right)$ for training [15], which is often impractical.

More fundamentally, current methods exhibit a key limitation: they do not simultaneously achieve (i) separation of overlapping components from the same class distribution, (ii) explicit reconstruction of each component, and (iii) training using only clean, non-overlapping samples. Attention-based classification models typically operate directly on mixtures without isolating individual components, while reconstruction-based methods rely on mixed–clean training pairs or large combinatorial datasets. As a result, they struggle in scenarios where overlapping observations are unavailable during training or where the number of possible mixtures is prohibitively large.

To address this gap, we propose a conceptually simple and modular attentional framework, Classification and Reconstruction of Overlapping Patterns (CROP). CROP leverages attention mechanisms to exploit structural regularities in the data, integrating bottom-up classification with top-down generative reconstruction within a recurrent inference loop, thereby enabling progressive separation and identification of overlapping patterns. Unlike prior approaches, it operates using only clean training samples, while explicitly reconstructing and disentangling individual components drawn from the same distribution, differing from blind source separation (which relies on statistical independence) and standard multi-object classification (which does not explicitly reconstruct individual components). This design promotes interpretability, reduces training complexity, and avoids reliance on combinatorial mixture datasets.

The contributions of this paper are:

- **Recurrent classification–reconstruction framework**. We propose an iterative inference procedure that alternates between bottom-up classification and top-down generative reconstruction to progressively separate and classify overlapping patterns.
- **Attention-guided iterative separation**. The framework incorporates saliency- and priority-driven estimates that guide masking and reconstruction, enabling the sequential extraction of two overlapping signals from a mixture.
- **Clean-only training paradigm**. The generative component is trained exclusively on clean samples, eliminating the need for paired mixed–clean training data typically required by supervised separation methods and difficult to obtain in practice.
- **Integration of discriminative and generative models**. The approach combines a convolutional classifier with a conditional variational autoencoder within a recurrent inference loop that jointly supports classification and reconstruction.
- **Proof-of-concept evaluation**. Experiments on overlapping handwritten digits and structured interference demonstrate the feasibility of the proposed framework for jointly separating and classifying overlapping patterns.

## Methodology

In this section, we introduce a method for reconstructing and classifying two signals from overlapping measurements. This method utilizes a neural network and an iterative procedure that draws inspiration from attention mechanisms in the human brain, without requiring mixed training data.

### Problem formulation

A noisy observation $\tilde{x}$ is often modeled as a transformation of an underlying noiseless signal x, where the reconstruction task consists of recovering x from $\tilde{x}$. Within a Bayesian framework, x is treated as a random variable with prior density 𝑝(x), and the observation $\tilde{x}$ is generated through a stochastic transformation $\tilde{x}\sim 𝑞(\tilde{x}\;|\;x)$.

**Fig 1:**
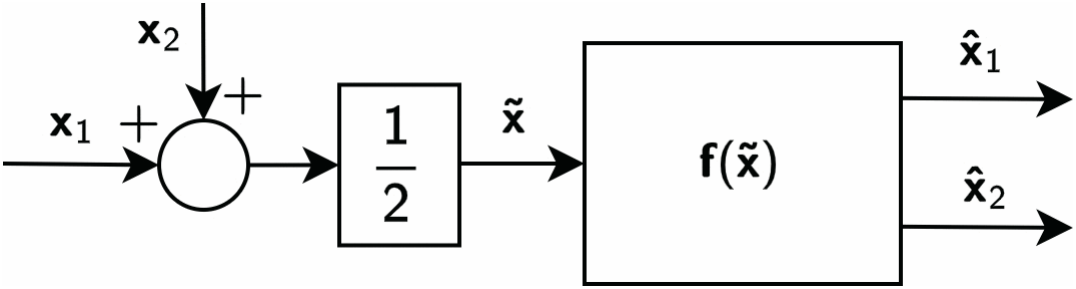
Classification or reconstruction from the scaled addition of two signals.

However, in the setting considered in this work, the observation does not arise from a generic noise process. Instead, it corresponds to the superposition of two structured signals drawn from the same distribution. Specifically, let $x_{1}\sim p_{X_{1}}$ and $x_{2}\sim p_{X_{2}}$ denote two underlying signals. The observed signal $\tilde{x}$ is then modeled as


$$\begin{array}{c} \tilde{x}=(1-\alpha )x_{1}+\alpha x_{2}, \end{array}$$ (1)

where α controls the relative contribution of each component. If both signals originate from the same stationary random process, then $p_{X_{1}}(\cdot )=p_{X_{2}}(\cdot )$. In particular, if α=1/2, the mixed signal is


$$\begin{array}{c} \tilde{x}=\frac{1}{2}(x_{1}+x_{2}), \end{array}$$ (2)

which results in an effective signal-to-noise ratio (SNR) of 0 dB under the assumption of equal power and uncorrelated components, where each signal can be interpreted as interference for the other (Fig 1).

When signal separation is required, ${\hat{x}}_{1}$ and ${\hat{x}}_{2}$ denote the reconstructed signals, and this task is more related to the well-known two-source separation or demixing problem. Alternatively, when the goal is to classify both signals into *k* classes, ${\hat{x}}_{1}$ and ${\hat{x}}_{2}$ can be interpreted as ${\hat{c}}_{1}$ and ${\hat{c}}_{2}$, representing the estimated class labels corresponding to the ground-truth labels $c_{1}$ and $c_{2}$, respectively. These labels are typically encoded as one-hot vectors, i.e.,


$$\begin{array}{c} c_{1},c_{2}\in {𝒞}^{k}=\backslash \{(c^{1},\ldots ,c^{k})\;|\;c^{i}\in \{0,1\},\;\sum_{i=1}^{k}c^{i}=1\backslash \}. \end{array}$$ (3)

### The proposed model

In order to process visual signals, we define $x_{1}$ and $x_{2}$ as the representations of two different objects sharing the same background, which are transparently mixed into a single scene $\tilde{x}$. Since the goal is to reconstruct and classify both objects, and given that the human brain efficiently performs such tasks, we propose an attention-inspired mechanism that combines bottom-up saliency with top-down object-oriented selection.

Although the full neural mechanisms remain unclear, numerous visual phenomena suggest that the brain’s predictive capacity plays a critical role in object identification and reconstruction. When we partially observe an object, we can often categorize it without perceiving all its details. Furthermore, if asked to draw the object, our reconstruction would typically be biased by the internal model we inferred.

Based on this reasoning, we propose a model that combines bottom-up feature categorization to construct a saliency map with a subsequent top-down object classification to iteratively refine the object reconstruction. In line with recent findings [16,17], our model leverages the idea that both stimulus-driven and goal-directed attention can be modeled by conditioning the responses of neural networks with categorical biases.

**Fig 2:**
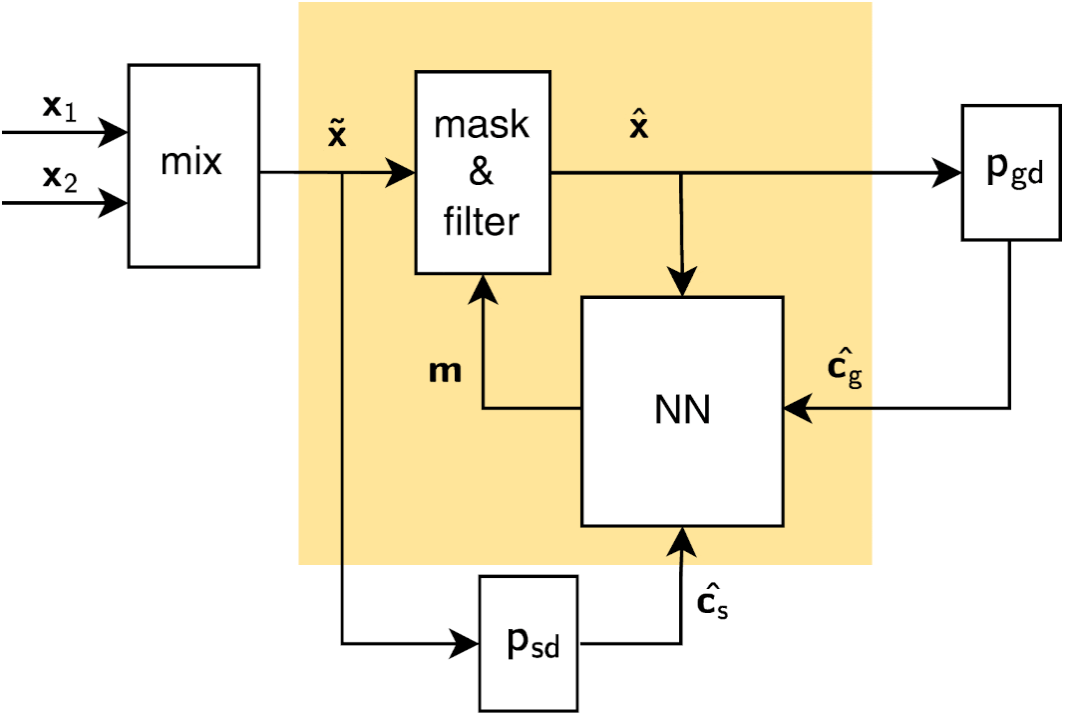
CROP model. The model includes bottom-up and top-down prediction–attention blocks ($p_{sd}$ and $p_{gd}$, respectively), a masking filter, and the conditional neural network “NN”. The input to the model is the mixed signal $\tilde{x}$.

Accordingly, conditional bottom-up and top-down inference processes assist the generative reconstruction of the object that best explains the observation. Specifically, we propose to include a bottom-up saliency-coded function $p_{sd}$ to produce an initial categorization ${\hat{c}}_{s}$ of the most recognizable signal $x_{i}^{*}$ from the mixed scene $\tilde{x}$ (Fig 2).

Recent studies have explored the interplay between visual and cognitive networks. Gong and Liu [18], using functional Magnetic Resonance Imaging (fMRI) data, observed a gradient of attentional biases—ranging from weaker biases in the visual network to stronger biases in the fronto-parietal network—suggesting a dimensional reduction from sensory processing to higher-level cognitive encoding of attentional priority.

Accordingly, we propose incorporating a neural network “NN” with a bottleneck to account for such dimensional reduction. The network is originally trained with samples of $x_{1}$ and $x_{2}$, but never with the mixed signal $\tilde{x}$. It then processes $\tilde{x}$, conditioned on ${\hat{c}}_{s}$, to generate a saliency map. To achieve this, we employ an appropriate squashing function which generates a mask m that emulates the salience mechanisms observed in early sensory neural processing in animals, resembling a winner–take–all decision process [19,20]. This saliency map is then used to mask and filter the original signal $\tilde{x}$, passing through those regions that are more consistent with the saliency map, thereby producing an initial estimate $\hat{x}$ of the filtered object.

Melloni et al. [21] identified distinct roles for human visual areas: V1 primarily represents bottom-up signals, V2 responds to top-down modulations, and hV4 integrates both. They showed that top-down control from the frontal eye fields and lateral occipital cortex enhances bottom-up salience, while the left intraparietal sulcus suppresses distracting bottom-up signals. These findings underscore the brain’s flexible regulation of bottom-up and top-down attention, and motivate the inclusion of a second, more goal-directed priority-map function $p_{gd}$ to obtain a new classification ${\hat{c}}_{g}$ of the filtered signal $\hat{x}$. This classification reinforces or corrects the original categorization ${\hat{c}}_{s}$ obtained from the mixed signal $\tilde{x}$.

The same neural network then constructs a new priority map m by processing the filtered signal $\hat{x}$, now conditioned on ${\hat{c}}_{g}$. This updated map is again applied to the original mixed signal $\tilde{x}$, and the process is repeated iteratively, progressively enabling the separation and classification of one of the two signals.

Since the ultimate goal is to separate both superposed signals, the process can be extended by suppressing the previously reconstructed signal from the original mixture to obtain a coarse estimate of the remaining signal. This residual is then classified by $p_{gd}$ into a new ${\hat{c}}_{g}$ corresponding to the alternate object, which in turn conditions the neural network to generate a new mask for $\tilde{x}$. The entire cycle is then repeated.

Although the bottom-up salience-based categorization ${\hat{c}}_{s}$ is applied only once, to select the most prominent object in the image, the top-down classification is alternated so that attention is directed to one object at a time. After several such cycles for each signal, we obtain progressively refined reconstructions together with their corresponding classifications.

This alternating process provides a two-fold advantage: it not only enables the sequential reconstruction of both signals, but also improves the reconstruction of each signal by exploiting information gained while reconstructing the other.

Each block can be modeled using different approaches; however, we chose to use neural networks to model not only the masking process but also the bottom-up and top-down mechanisms. Although several neural network architectures could be considered, in this work we evaluate a model that includes:

- a Conditional Variational Autoencoder (CVAE) [22],
- Convolutional Neural Network (CNN) classifiers for label prediction (*Predictors* — $p_{sd}$ and $p_{gd}$), and
- a recurrent inference process to iteratively reconstruct and classify both signals.

A Variational Autoencoder (VAE) consists of an *encoder* and a *decoder* [23]. The encoder maps the input into a *latent variable* z sampled from a latent space, typically a low-dimensional manifold. Two neural networks are usually employed: the first computes the latent variable, $z=q_{\phi }(x)$, and the second estimates the reconstruction, $\hat{x}=p_{\theta }(z)$, where $q_{\phi }$ and $p_{\theta }$ are deterministic functions parameterized by ϕ and θ, respectively. For simplicity of notation, we will also use $q_{\phi }$ and $p_{\theta }$ to denote the corresponding probability density functions.

Since the main objective is to obtain $\hat{x}≃x$, the negative log-likelihood of x is commonly minimized using stochastic gradient descent. A variational lower bound of $\log p_{\theta }(x)$ for the VAE, known as the Evidence Lower Bound (ELBO), is given by


$$\begin{array}{c} {ℒ}_{\theta ,\phi }(x)={𝔼}_{z\sim q_{\phi }(z|x)}[\log p_{\theta }(x\;|\;z)]-D_{KL}\;\left[q_{\phi }(z\;|\;x)\;\|\;p_{\theta }(z)\right], \end{array}$$ (4)

where the second term represents the Kullback–Leibler divergence (KLD) between the approximate posterior and the prior distribution. Thus, the ELBO depends on the prior $p_{\theta }(z)$ and the models $p_{\theta }(x\;|\;z)$ and $q_{\phi }(z\;|\;x)$. During training, the parameters θ and ϕ are optimized to maximize the ELBO, thereby improving the approximation of $p_{\theta }(x)$.

**Fig 3:**
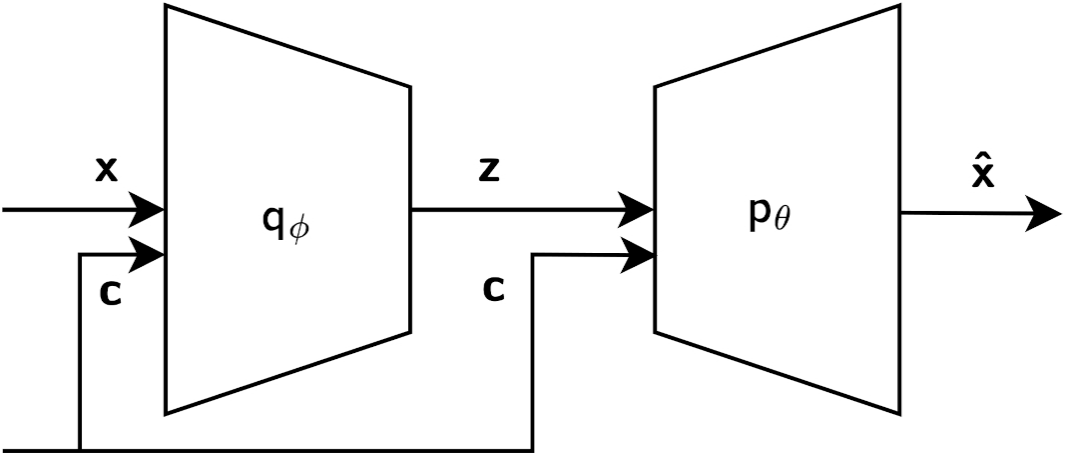
A possible implementation of a CVAE model.

A CVAE can be constructed by introducing a condition c, where $c^{\left(i\right)}$ represents the label (or other relevant attribute) associated with each data sample $x^{\left(i\right)}$. The condition $c^{\left(i\right)}$ is input to the encoder alongside $x^{\left(i\right)}$, while the decoder receives both the latent variable $z^{\left(i\right)}$ and the condition $c^{\left(i\right)}$ (Fig 3). When both the encoder and the decoder are conditioned on c, the ELBO for the CVAE is given by:


$$\begin{array}{cc} {ℒ}_{\theta ,\phi }^{\text{CVAE}}(x,c) & ={𝔼}_{z\sim {𝑞}_{\phi }(z|x,c)}[\log{𝑝}_{\theta }(x\;|\;z,c)]-D_{KL}[{𝑞}_{\phi }(z\;|\;x,c)\;\|\;{𝑝}_{\theta }(z\;|\;c)]. \end{array}$$ (5)

#### Training.

During training, both x and c are typically known. Thus, the parameters θ and ϕ can be optimized by maximizing the ELBO using the reparameterization trick [23]. By choosing a class-independent prior ${𝑝}_{\theta }(z\;|\;c)={𝑝}_{\theta }(z)=𝒩(0,𝐈)$, the latent variable z is encouraged to capture only residual, intra-class variability, while the class label c explicitly guides both the encoder and decoder. In this sense, c acts as an attentional signal that biases the model toward class-specific structure, whereas z represents finer-scale variations within each class.

Suppose that the condition c can be estimated from the input signal x. A natural choice is to employ a neural network $f_{\xi }$ with parameters ξ to predict $\hat{c}=f_{\xi }(x)$ deterministically, where $\hat{c}$ is a vector representing the estimated condition (e.g., class probabilities). Alternatively, this network can be interpreted probabilistically as modeling a conditional distribution ${𝑝}_{\xi }(c\;|\;x)$, where c denotes the corresponding class indicator vector. The network can be trained offline using standard backpropagation to optimize ξ by approximating the mapping from x to c.

**Fig 4:**
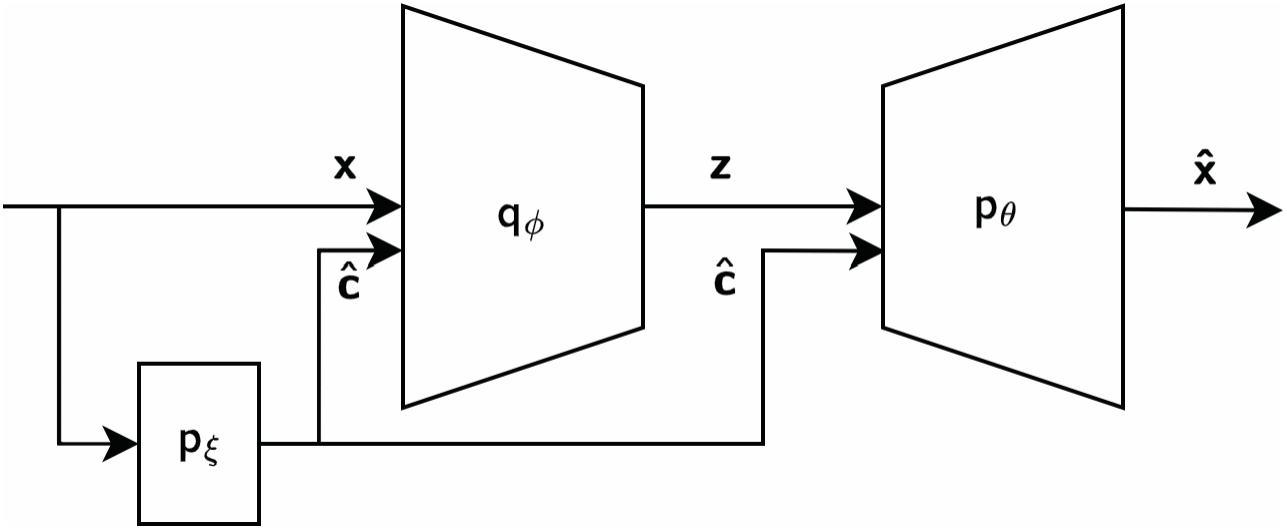
Alternative process for training the CVAE model using predicted class labels.

Since the predictor network is trained prior to the CVAE, both the encoder and decoder can be conditioned on the predicted label $\hat{c}$ (Fig 4).

Accordingly, the Evidence Lower Bound (ELBO) is modified as:


$$\begin{array}{cc} {ℒ}_{\theta ,\phi }^{\text{CVAE}}(x,\hat{c}) & ={𝔼}_{z\sim {𝑞}_{\phi }(z|x,\hat{c})}[\log{𝑝}_{\theta }(x\;|\;z,\hat{c})]-{𝐷}_{KL}[{𝑞}_{\phi }(z\;|\;x,\hat{c})\;\|\;{𝑝}_{\theta }(z\;|\;\hat{c})], \end{array}$$ (6)

where ${𝑝}_{\theta }(z\;|\;\hat{c})={𝑝}_{\theta }(z)=𝒩(0,𝐈)$ and $\hat{c}\sim {𝑝}_{\xi }(\hat{c}\;|\;x)$.

During testing, x is available while c is unknown. An estimate $\hat{c}$ can be obtained directly from x using the predictor ${𝑝}_{\xi }(c\;|\;x)$. This ensures that the true condition c is required only during training, allowing the model to operate autonomously without explicit label information at inference time.

#### Inference: Recurrent regression for filtering.

The proposed framework relies on two computational components: a classifier ${𝑝}_{\xi }(c\;|\;x)$ and a conditional generative model defined by the encoder–decoder pair ${𝑞}_{\phi }(z\;|\;x,c)$ and ${𝑝}_{\theta }(x\;|\;z,c)$. The classifier provides class estimates that guide the reconstruction process, while the generative model produces signal reconstructions conditioned on these estimates.

During inference, the classifier is evaluated at two stages within an iterative loop. When applied directly to the mixture $\tilde{x}$, it produces a saliency estimate ${\hat{c}}_{s}$, representing a bottom-up hypothesis about the most prominent pattern in the mixture. Conditioned on this estimate, the generative model samples $z\sim {𝑞}_{\phi }(z\;|\;\tilde{x},{\hat{c}}_{s})$ and produces a reconstruction $\hat{x}\sim {𝑝}_{\theta }(x\;|\;z,{\hat{c}}_{s})$.

A mask m is then derived from $\hat{x}$ and applied element-wise to the mixture $\tilde{x}$ to isolate the corresponding component. The extracted pattern is subtracted from the mixture to obtain the residual signal. The classifier is subsequently applied to the reconstructed signal, yielding a refined estimate ${\hat{c}}_{g}={𝑝}_{\xi }(\hat{x})$, which serves as a top-down update of the initial hypothesis. The attention-inspired mechanism is thus implemented as a continuous soft mask $m\in [0,1{]}^{n}$, where *n* denotes the dimensionality of the input signal. The mask constitutes the explicit variable that performs the filtering, enabling progressive extraction of individual patterns.

The masking function Mask(·) is defined as a deterministic element-wise transformation that converts the reconstructed signal into a soft attention map. Specifically, given a reconstruction ${\hat{x}}_{k}$, the mask is computed as


$$\begin{array}{c} m_{k}=\sigma \;\left(\beta \;({\hat{x}}_{k}-\tau )\right), \end{array}$$ (7)

where σ(·) denotes the sigmoid function applied element-wise, β>0 controls the slope, and τ is a bias term that determines the activation threshold. The resulting mask $m_{k}\in [0,1{]}^{n}$ emphasizes regions consistent with the reconstructed pattern while suppressing others. This mask is then applied element-wise to the input mixture $\tilde{x}$ to extract the corresponding component. This formulation can be interpreted as a soft winner–take–all mechanism.

The terms *saliency map* and *priority map* refer to the class estimates ${\hat{c}}_{s}$ and ${\hat{c}}_{g}$, respectively. The former is obtained directly from the mixture, while the latter is computed from the reconstructed signal. Although both correspond to evaluations of the same classifier ${𝑝}_{\xi }$, they operate on different inputs and serve distinct roles within the inference loop. Accordingly, the bottom-up stage refers to classification from the mixture, providing an initial hypothesis, whereas the top-down stage corresponds to classification of the reconstructed signal, refining this hypothesis. From a biological perspective, these stages resemble saliency-driven and reconstruction-guided processes; however, in implementation they are realized by a single classifier evaluated on different inputs.

The entire procedure is repeated iteratively, progressively improving the reconstruction of both patterns while updating their corresponding class estimates, ultimately leading to the classification of both signals. (Algorithm 1 in Table 1).

**Table 1:** Algorithm 1: CROP inference pseudo-code.

Input: Mixed signal $\tilde{x}$	
1. k←0	
2. ${\hat{x}}_{0}\leftarrow \tilde{x}$	
3. ${\hat{c}}_{0}\leftarrow p_{\xi }({\hat{x}}_{0})$	
4. **repeat**	
a) $z\sim q_{\phi }(z|{\hat{x}}_{k},{\hat{c}}_{k})$	
b) ${\hat{x}}_{k}\leftarrow p_{\theta }(x|z,{\hat{c}}_{k})$	// reconstructed pattern
c) $m_{k}\leftarrow \text{Mask}({\hat{x}}_{k})$	
d) ${\hat{x}}_{k}\leftarrow \tilde{x}⊙m_{k}$	// filtered pattern
e) k←k+1	
f) ${\hat{x}}_{k}\leftarrow \tilde{x}-{\hat{x}}_{k-1}$	// remaining pattern
g) ${\hat{c}}_{k}\leftarrow p_{\xi }({\hat{x}}_{k})$	
5. **until** FinishCondition()	
6. **return** ${\hat{x}}_{k-1},{\hat{c}}_{k-1},{\hat{x}}_{k},{\hat{c}}_{k}$	

Accordingly, the last two reconstructions $\left({\hat{x}}_{k-1},{\hat{x}}_{k}\right)$ and class estimates $\left({\hat{c}}_{k-1},{\hat{c}}_{k}\right)$ should provide good approximations to $\left(x_{1},x_{2}\right)$ and $\left(c_{1},c_{2}\right)$, respectively. It is important to note that the order $\left(x_{1},x_{2}\right)$ or $\left(x_{2},x_{1}\right)$ is irrelevant, since each signal acts as noise for the other, as long as the ordering is consistent with the corresponding class labels $\left(c_{1},c_{2}\right)$ or $\left(c_{2},c_{1}\right)$.

## Case study: Overlapping digit separation

Because our model is not trained using paired clean–mixed data $\left(x,\tilde{x}\right)$, a direct comparison with state-of-the-art approaches that explicitly rely on such paired information for joint classification and reconstruction [24] would not be entirely fair. However, as a proof of concept, and to enable comparison with other algorithms addressing the same classification and reconstruction tasks, we instead adopt as a baseline the models proposed by Mengu et al. [3], which were developed for the simultaneous classification and reconstruction of two spatially overlapping phase images. For consistency, we use the same dataset as in their work: the MNIST database [25], consisting of 28×28 grayscale images of handwritten digits.

### Experimental setting

Since both signals originate from the same distribution, we have $x_{1},x_{2}\in {𝒳}^{n}$ with corresponding labels $c_{1},c_{2}\in {𝒞}^{k}$. Therefore, a single CVAE can be trained to reconstruct both ${\hat{x}}_{1}$ and ${\hat{x}}_{2}$, and a single CNN-based classifier (hereafter referred to as the *Predictor*) can be trained to estimate ${\hat{c}}_{1}$ and ${\hat{c}}_{2}$.

CNNs have been shown to successfully solve core object-classification tasks by modeling the processing performed in the inferotemporal cortex (ITC); additional feedback connections have been proposed for more challenging scenarios [26–29]. In particular, Kornblith and Tsao [30] suggested that CNNs learn features resembling those represented in the ITC, as CNNs trained to predict ITC activity exhibited improved categorization performance. More recently, Rose and Ponce [31] argued—based on recordings from ventrolateral prefrontal cortex (vlPFC) neurons in monkeys—that computational models inspired by the prefrontal cortex should partially incorporate convolutional processing.

Motivated by these findings, we trained a shallow CNN-based *Predictor* for label estimation. The network receives 784 input values (corresponding to 28×28 pixel images) and consists of two convolutional layers with 32 and 64 filters, respectively, each with a 3×3 kernel and stride of 2. Each convolutional layer is followed by batch normalization. After flattening and applying dropout, the network terminates in a 10-neuron softmax layer that outputs class probabilities.

Similarly, we implement a shallow CVAE consisting of a fully connected two-layer *Encoder* and a two-layer *Decoder*. The *Encoder* takes as input the 784 image pixels concatenated with the 10-dimensional one-hot label vector, followed by a hidden layer of 512 neurons. Its output comprises two 256-dimensional vectors representing the mean and log-variance of the latent variables z. Although lower latent dimensions were evaluated with comparable reconstruction performance, we selected 256 to improve representational capacity and better capture fine-grained variations in the reconstructed patterns. The Decoder receives z concatenated with the labels, followed by a hidden layer of 512 neurons and an output layer of 784 neurons to reconstruct the image. Since each mixed input is processed sequentially within the iterative inference procedure, the same decoder is reused across iterations to refine reconstructions of the separated components.

We use a sigmoid activation at the *Decoder* output to model salience and priority maps that enhance or suppress different image regions. While other bounded squashing functions could be used, we selected the sigmoid due to its stable behavior in this setting. Its parameters were determined via grid search: the bias term was explored in the range [0.1, 0.6] and the slope in [8, 30], using reconstruction accuracy as the evaluation criterion. The selected values were a bias of 0.2 and a slope of 22, which provided the best trade-off between reconstruction fidelity and separation performance in this scenario.

We used the MNIST dataset [25], consisting of 60,000 handwritten digits for training and 10,000 for testing. To ensure a fair comparison, the training set for both the *Predictor* and the CVAE comprised 55,000 clean images taken from the original training set, following the setup in Mengu et al. [3]. The key distinction is that, whereas Mengu et al. generated roughly 550 million overlapping digit samples by pairing MNIST images, our method does not require such a large synthetic dataset for training. As in [3], 10,000 images were reserved as the standard test set (T1). In addition, we constructed a second test set, T2, containing 10,000 new samples generated by randomly overlapping two distinct digits (without replacement) from T1. Thus, T2 is used to assess the CROP model’s demixing and classification performance on previously unseen overlapping patterns.

The *Predictor* was trained using the categorical cross-entropy loss. The CVAE parameters were learned by minimizing a combined loss consisting of the mean squared error (MSE) between input and reconstructed images and the Kullback–Leibler divergence between the *Encoder* output and 𝒩(0,I). Each epoch used all 55,000 clean MNIST training images sampled without replacement. The *Predictor* and CVAE were trained sequentially using only clean images; importantly, neither network was ever exposed to overlapping images during training.

Two criteria were used to evaluate performance:

1. **Classification accuracy.** The *Predictor* output used for conditional estimation was compared against the ground-truth labels of the two original digits. Two indices were reported: (a) both predictions correct, and (b) only one prediction correct.
2. **Reconstruction quality.** Two standard image similarity metrics were employed to facilitate comparison with existing methods: (a) the Structural Similarity Index Measure (SSIM), and (b) the Peak Signal-to-Noise Ratio (PSNR).

The CROP algorithm was implemented in Python 3.10.12 and TensorFlow 2.15.0 (Google Inc.). The Adam optimizer was used with default parameters, a learning rate of 0.001, and a batch size of 128 for all networks. All experiments were run on the standard CPU environment provided by TensorFlow (Intel^®^ Xeon^®^ CPU @ 2.20 GHz with 12 GB RAM).

All model architectures used in this work are described in the methodology section, while the training configurations were explained in this section to facilitate reproducibility. The classifier and conditional generative model architectures were selected empirically to balance reconstruction quality and computational complexity rather than to optimize biological plausibility. All experiments were conducted using models trained exclusively on clean samples. During inference, the iterative procedure was executed until a predefined number of steps as a stopping criterion.

### Results on overlapping digit separation

After training the CROP model—specifically, once both the CVAE and the *Predictor* were trained exclusively on clean images—the testing phase was conducted using the T2 dataset, which consists of previously unseen pairs of overlapping handwritten digits.

**Fig 5:**
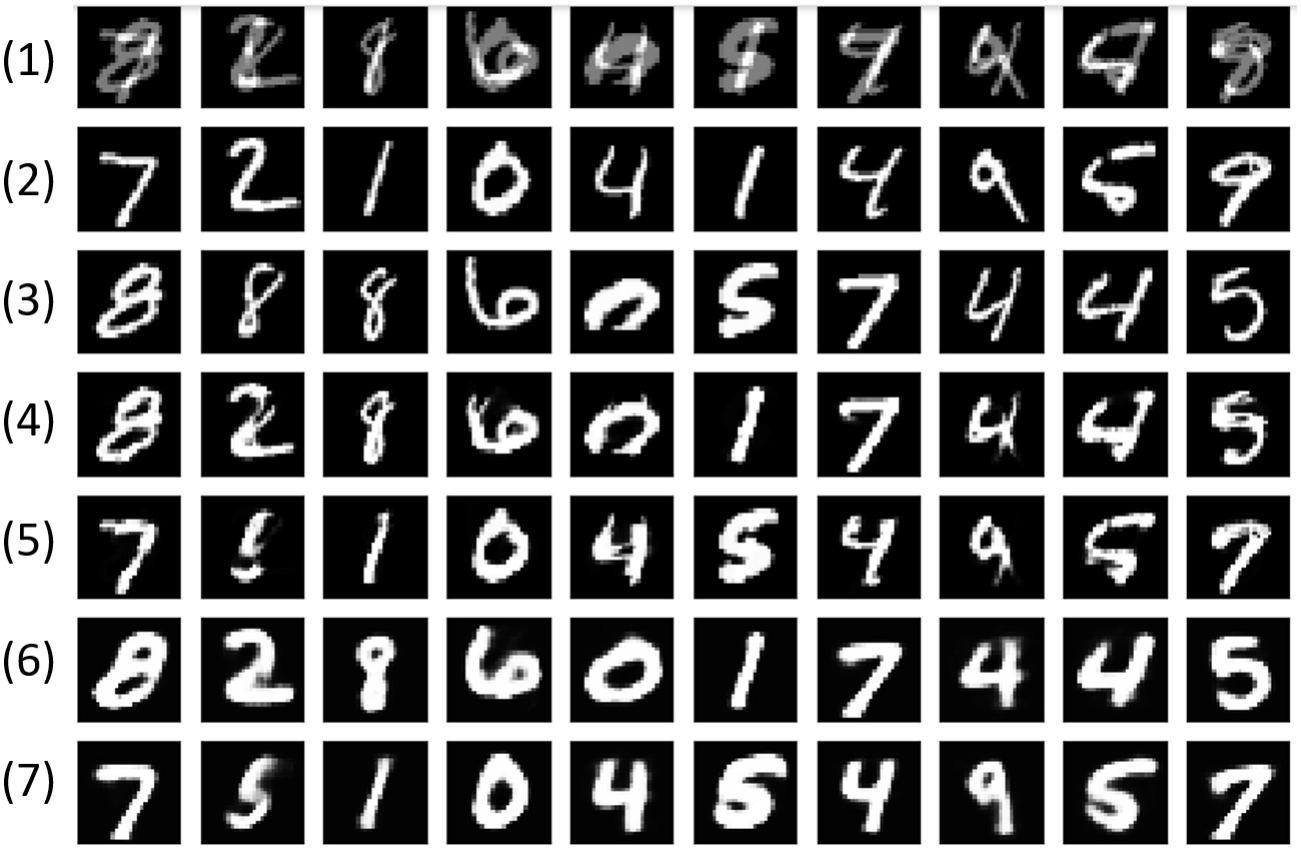
CROP – Test. Uncurated examples of the mixed signals are shown in the first row. The corresponding original (unmixed) signals are displayed in the second and third rows. The fourth and fifth rows present the reconstructions obtained by the CROP algorithm, shown in the order they were generated. Finally, the sixth and seventh rows display the corresponding masking outputs produced during the reconstruction process.

Fig 5 presents examples of both successful and unsuccessful reconstructions from the T2 test set. Although not all digits are correctly recovered, many reconstructions exhibit high visual fidelity. Comparing the original digits (rows 2 and 3) with their corresponding reconstructions (rows 4 and 5) reveals strong similarities. For example, the “8” in the first column (rows 3 and 4) is nearly identical in both cases. However, some failure cases occur; for instance, in the last column, the “9” in row 2 is reconstructed as a “7” in row 5.

Rows 6 and 7 display the final masks produced by the CROP algorithm, which guide the filtering process. Notably, these masks can also be interpreted as smooth approximations of the underlying digits.

In these experiments, a step denotes one full iteration of the separation procedure described in Algorithm 1, applied sequentially to the two digits. After the initial saliency estimation, each step consists of reconstruction, masking, and priority update operations for both digits. The iteration counts reported in the experiments indicate the number of steps required for CROP to reach the reported accuracy levels, and are used as a measure of convergence speed when comparing with other methods, rather than a fixed requirement of the algorithm.

For evaluation on labeled test data, we imposed a stopping criterion by terminating the algorithm after a fixed number of inference steps. This choice was made solely to bound the experimental runtime and to enable consistent comparison across methods. All reported results are obtained using a fixed number of iterations. In practical deployments with unlabeled mixtures, the algorithm may instead run for a predefined number of steps or rely on an internal convergence criterion.

To assess performance robustness, the algorithm is evaluated over 20 independent runs, each consisting of 50 inference steps. Classification accuracy is averaged across runs, while SSIM and PSNR are computed from the reconstructions obtained at the final inference step.

The accuracy for correctly classifying both digits increased from (54.04±0.19)% after a single step to (79.62±0.22)% after five steps, and (86.97±0.31)% after 50 steps. Across all 50 steps, the standard deviation remained below 0.5%, and the difference between the maximum and minimum accuracy over the 20 runs was always below 2%. These results indicate that errors in the initial step have limited impact as the recurrent inference progresses, with the iterative procedure consistently converging to similar solutions. These results also suggest robustness to the domain shift between clean training data and mixed inputs at inference time.

**Fig 6:**
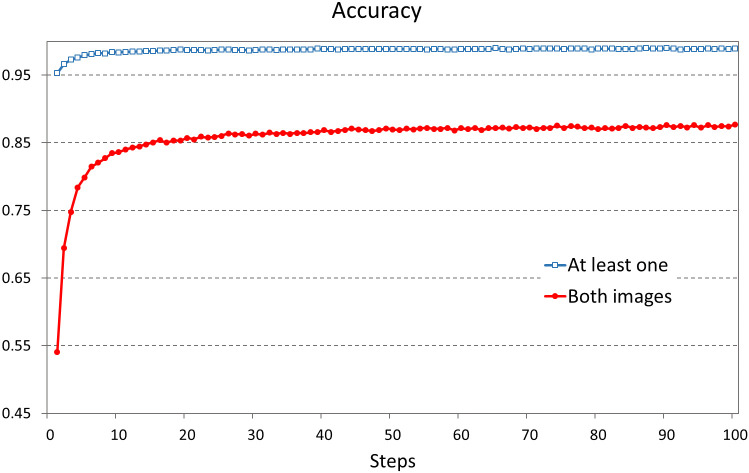
Accuracy evolution of the CROP algorithm when recognizing one or both digits from T2 dataset.

The final accuracy reached 87.69% on average at convergence after 100 steps; notably, more than 90% of this value was achieved after only 5 steps (Fig 6), indicating fast convergence in the early iterations.

As a complementary indicator, we also report the accuracy when at least one digit is correctly classified, although this metric does not represent full task success. Under this criterion, accuracy exceeded 94% after one step, surpassed 97% after three steps, and reached 98.90% at the end of the experiment.

When evaluated on the T1 dataset, consisting of clean single-digit images, the model achieved 98.25% accuracy after an average of 10 inference steps, consistent with the absence of overlapping patterns.

Table 2 presents the classification accuracy of the CROP algorithm across two test sets. As a reference, we consider four Diffractive Deep Neural Networks (D^2^NN) proposed by Mengu et al. [3]: D^2^NN-D1, D^2^NN-D1d, D^2^NN-D2, and D^2^NN-D2d, which differ in detector configurations and class encoding schemes at the output plane. These models are designed to represent all possible class combinations of overlapping phase objects at the input. Their reported blind-test accuracies for classifying two overlapping handwritten digit phase images (T2) range from 82.61% to 85.82%, while for the T1 test set it reached 94.20%, after training for 20,000 epochs on approximately 550 million digit combinations with corresponding labels. In contrast, our approach requires only 55,000 single-digit samples and fewer than 100 training epochs. While the two approaches differ in architecture and training assumptions, this comparison provides a useful reference point to highlight the relative data efficiency of the proposed method.

**Table 2:** Accuracy of the CROP and other four baseline algorithms on two test sets.

	Test set T2	Test set T1
Solution	Accuracy [%]	Accuracy [%]
D^2^NN-D1	82.70	90.59
D^2^NN-D1d	85.82	93.30
D^2^NN-D2	82.61	93.38
D^2^NN-D2d	85.22	94.20
CROP	**87.69**	**98.25**

Besides the classification accuracy on the T2 test set, Table 3 shows the reconstruction quality for all pairs of images. To assess the quality of the images reconstructed by the CROP algorithm, we used the same metrics proposed by Mengu et al. [3]: (a) SSIM and (b) PSNR, achieving an SSIM of 0.79 and a PSNR of 17.98 dB (where higher values indicate better reconstruction quality). It is likely that the classification accuracy and image quality are correlated, as the iterative process aims to improve both outcomes jointly.

**Table 3:** Reconstruction quality (SSIM and PSNR) of the CROP algorithm and four baseline models on the T2 test set (mean ± standard deviation).

Solution	Accuracy [%]	SSIM	PSNR [dB]
D^2^NN-D1	82.70	0.52 ± 0.12	15.09 ± 2.32
D^2^NN-D1d	85.82	0.57 ± 0.10	16.02 ± 2.21
D^2^NN-D2	82.61	0.49 ± 0.10	14.55 ± 2.17
D^2^NN-D2d	85.22	0.57 ± 0.12	15.60 ± 2.37
CROP	**87.69**	**0.79 ± 0.10**	**17.98 ± 2.59**

**Fig 7:**
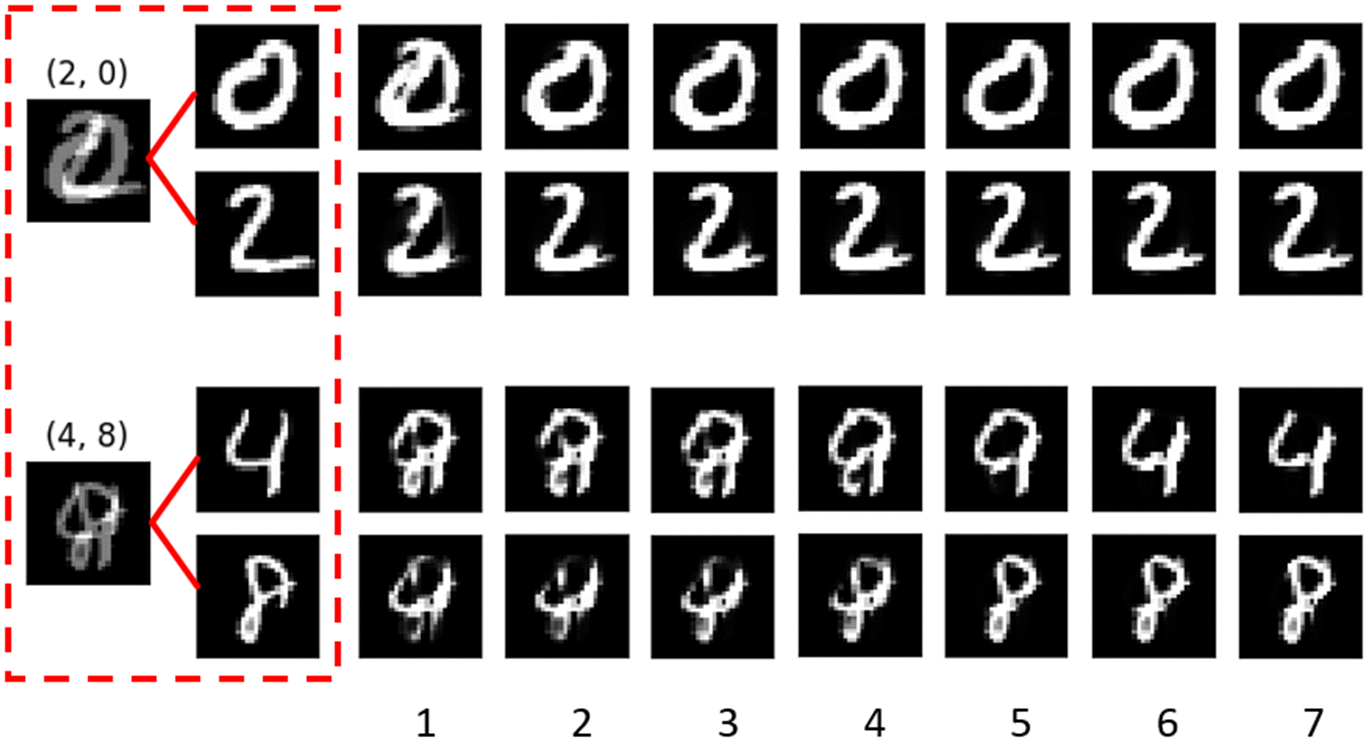
Evolution of the reconstructed images. The red dotted block shows the overlapping digits and their ground-truth decompositions. The seven columns to the right illustrate the first seven reconstruction steps. The top rows show rapid convergence, whereas the bottom rows depict more challenging cases.

Although our framework relies on standard deep neural network components, the iterative inference process exposes intermediate variables — including class estimates, reconstructed signals, and masks — that provide a transparent view of how the model progressively isolates each pattern from the mixture. This step-by-step reconstruction makes it possible to visually inspect the contribution of each iteration and to understand how classification hypotheses guide the separation process. In this sense, the model offers a form of process-level interpretability despite relying on deep generative and discriminative components. Fig 7 illustrates the intermediate reconstructions produced during the iterative process.

These results demonstrate that the proposed framework can jointly perform separation and classification using only clean training samples and substantially fewer data than competing approaches.

## Case study: Structured interference separation

Another application of the CROP algorithm is the separation of a signal from its superposition with structured interference. When both components are reconstructed, the process is referred to as demixing; when only the target signal is recovered and the interference is suppressed, the process corresponds to denoising.

We follow the same experimental setup as in the previous case. The MNIST dataset is used as the target signal, while structured interference is generated using random vertical and horizontal lines. In this setting, both components are assumed to be independent.

The interference patterns are treated as an additional class. Accordingly, the predictor and the CVAE are trained on eleven balanced classes, using 60,500, 5,500, and 11,000 images for training, validation, and testing, respectively. During inference, 10,000 structured interference images—unseen during training—are superimposed onto samples from the T1 dataset.

Performance is evaluated over 20 runs of 10 inference steps each. Classification accuracy is averaged across runs, while SSIM and PSNR are computed using the reconstructions from the final inference step.

### Interference: Vertical and horizontal lines

In these experiments, the interference consists of vertical and horizontal lines placed at random positions across the MNIST images. Two configurations are considered: (i) two coarse vertical and two coarse horizontal lines (each three pixels wide), and (ii) seven vertical and seven horizontal thin lines (each one pixel wide).

The predictor is trained using early stopping with a maximum of 100 epochs in both cases. The CVAE is trained for 50 epochs in the coarse-line configuration and 100 epochs in the thin-line configuration.

The classification accuracy reported in Table 4 indicates that most of the eleven classes (digits and interference) are correctly identified. The high SSIM and PSNR values further suggest that both components are reconstructed with low distortion, indicating the capability to distinguish between signal and structured interference while preserving reconstruction quality.

**Table 4:** Reconstruction quality (SSIM and PSNR) and classification accuracy for the T1 test set with overlapping line interference (mean ± standard deviation).

Interference	SSIM	PSNR [dB]	Accuracy [%]
Coarse lines	0.83 ± 0.09	27.52 ± 4.36	86.93 ± 0.20
Thin lines	0.84 ± 0.07	27.15 ± 1.92	88.86 ± 0.29

**Fig 8:**
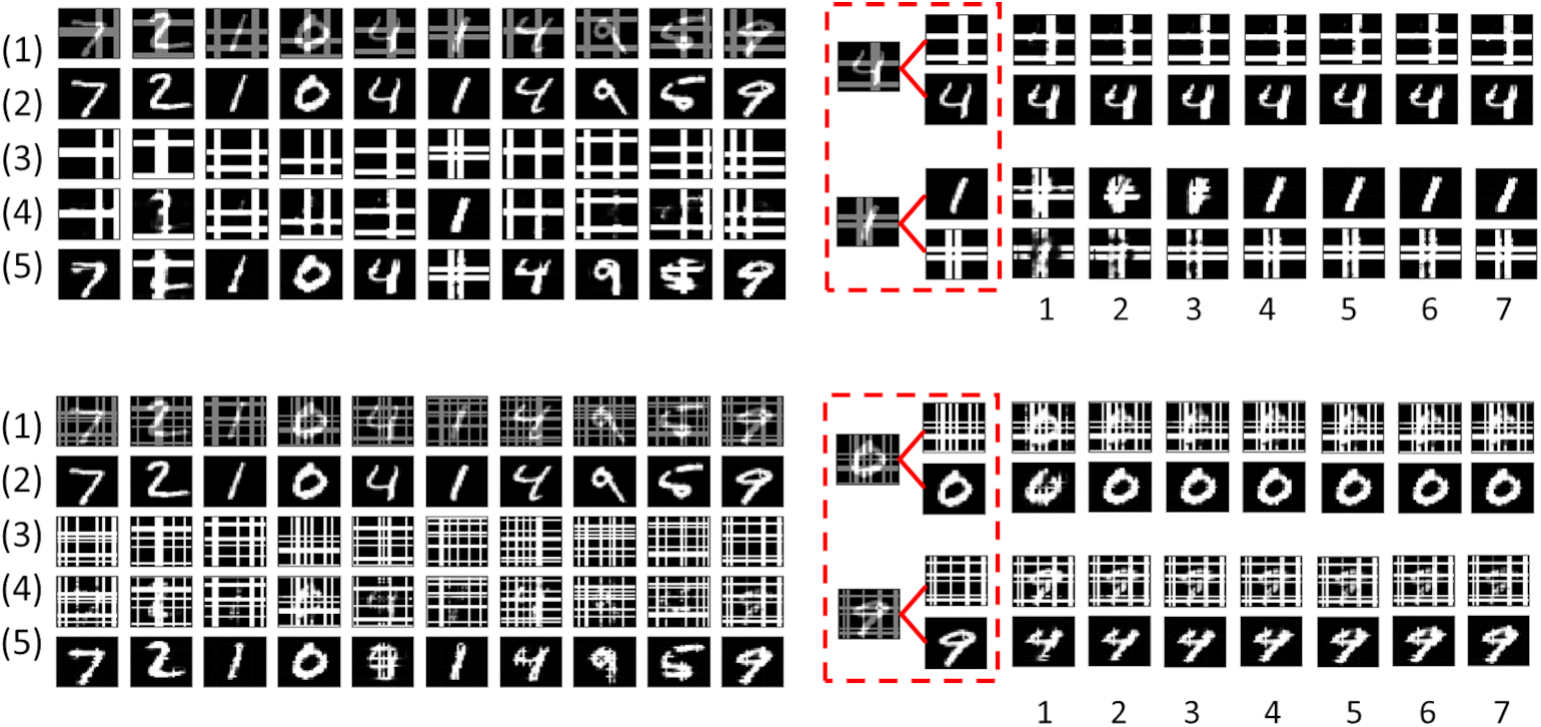
Reconstruction of digits and interference lines. Top left (coarse) and bottom left (thin): Uncurated examples of the mixed signals are shown in row (1). The corresponding original (unmixed) signals are displayed in rows (2) and (3). The reconstructions obtained by the CROP algorithm in rows (4) and (5), are shown in the order they were generated. Top right and bottom right: The red dotted block shows the overlapping images and their ground-truth decompositions. The seven columns to the right display the first seven reconstruction steps. The top rows illustrate rapid convergence, whereas the bottom rows correspond to more challenging cases.

This behavior is illustrated in Fig 8, which shows ten random samples after the first ten inference steps. In most cases, both the signal and the interference are well reconstructed, although partial recovery is observed in some instances. The same figure also presents the evolution of two samples over the first seven inference steps, highlighting that convergence rates vary with the degree of overlap and structural complexity of the mixed signal.

## Discussion

Recognizing and reconstructing overlapping patterns drawn from the same probability distribution remains a challenging problem for demixing systems, where the distinction between “signal” and “noise” is inherently ambiguous. Traditional approaches must address several difficulties, including: (i) the need for large datasets of clean and mixed samples, (ii) long training times, (iii) accurate separation and reconstruction of individual components, and (iv) reliable classification performance. Additional considerations such as computational complexity, inference time, adaptability, and interpretability further influence the choice of method.

A variety of approaches have addressed these challenges. Early work by Vincent et al. [32] introduced the Denoising Autoencoder (DAE), which relies on clean–noisy pairs $\left(x,\tilde{x}\right)$. Chen and Srihari [33] extended this framework with feedback mechanisms, while Chen et al. [34] incorporated class labels (c), using triplets $\left(x,\tilde{x},c\right)$ to jointly perform reconstruction and classification. Despite their effectiveness, these methods depend on large datasets of mixed samples, resulting in increased training complexity.

More recent approaches explore alternative strategies. The Memory for Latent Representations (MLR) model [35] encodes attributes in a binding pool but lacks iterative feedback during inference. Diffractive optical networks [3] achieve competitive performance but require training on a combinatorial number of overlapping samples, raising scalability concerns. Similarly, the Latent Autoregressive Source Separation (LASS) algorithm [24] separates signals without class labels but relies on paired datasets and an additional classifier.

Attention-based models such as OCRA [29] and ORA [9] incorporate iterative refinement mechanisms. However, OCRA is trained on overlapping inputs, while ORA relies on an encapsulated classification strategy and requires weight updates at each iteration step.

In contrast, CROP addresses a more constrained but practically relevant setting: the separation and classification of two overlapping patterns from the same distribution using only clean training samples. The proposed framework integrates classification and reconstruction within a unified iterative loop, where class estimates guide a generative model to progressively isolate individual components. This design eliminates the need for combinatorial mixed datasets and reduces training complexity.

The generative model acts as a class-conditioned prior, enabling reconstruction even when the input mixture lies outside the training distribution. At inference time, the classifier provides an initial hypothesis that is iteratively refined through reconstruction and masking. This interaction between bottom-up estimation and top-down reconstruction enables progressive separation without requiring weight updates during inference.

This design yields practical benefits: CROP achieves competitive classification and reconstruction performance with significantly reduced training data and fewer training epochs. In our experiments, most of the final accuracy is reached within a small number of inference steps. Comparisons with diffractive optical networks [3] provide a useful reference point, although the two approaches differ in architecture and training assumptions.

The current formulation of the proposed framework was initially restricted to mixtures of two overlapping patterns drawn from the same distribution. However, extending the framework to demixing structured interference is relatively straightforward, as shown in the experiments. Effective learning requires that the images exhibit spatial correlation and are sampled from the same class distribution, allowing the neural networks to capture the dominant structural features. However, performance degrades when patterns from different classes exhibit high structural similarity, since the separation task becomes inherently ambiguous under such conditions. The iterative reconstruction process introduces additional computational cost and may generate artifacts that impact both classification accuracy and reconstruction quality. However, experimental results indicate that the proposed approach is robust to variations in initial conditions and consistently converges to similar solutions. Incorporating explicit error-correction mechanisms may further improve performance. The method also entails additional computational cost at inference due to its iterative nature; however, convergence is typically achieved within a small number of iterations.

The experimental setup assumes fixed mixing conditions and uncorrelated sources, which limits direct applicability to more general scenarios involving variable mixtures, higher numbers of components, or heterogeneous data distributions. Consequently, the results should be interpreted as a proof of concept demonstrating the feasibility of the proposed recurrent inference framework.

Future work will focus on extending the approach to more complex settings, including multiple overlapping components, higher-resolution data, and heterogeneous sources. Additional improvements may also be achieved by incorporating more advanced classification modules to address domain shifts and category shifts between domains [36,37] or exploring alternative generative architectures.

## Conclusion

In this work, we proposed a method for the classification and reconstruction of two overlapping patterns drawn from the same probability distribution, even under challenging conditions (e.g., SNR = 0 dB). The approach integrates a Conditional Variational Autoencoder with a recurrent inference procedure inspired by bottom-up saliency and top-down guidance, enabling iterative separation and classification using only clean training data. This avoids the need for combinatorial datasets of mixed patterns while reducing training complexity.

Experiments on overlapping MNIST digits show that CROP achieves 87.69% classification accuracy, reaching over 90% of its final performance within a few inference steps. The model also achieves a reconstruction quality of 17.98 dB PSNR and 0.79 SSIM, suggesting preservation of structural information.

We also performed experiments with structured interference on MNIST digits which show that both digits and interference patterns are generally well identified. The high SSIM and PSNR values further indicate that both components are reconstructed with low distortion, suggesting that the model effectively distinguishes between signal and structured interference.

Overall, the proposed framework couples discriminative and generative components within a unified iterative inference scheme, where intermediate masks and reconstructions provide insight into the separation process. This enables demixing from clean-only training data while maintaining competitive performance under the studied conditions. The results demonstrate the feasibility of iterative classification–reconstruction for overlapping pattern separation and provide a basis for further exploration of hybrid discriminative–generative inference models.
